# Direct exfoliation and dispersion of two-dimensional materials in pure water via temperature control

**DOI:** 10.1038/ncomms9294

**Published:** 2015-09-15

**Authors:** Jinseon Kim, Sanghyuk Kwon, Dae-Hyun Cho, Byunggil Kang, Hyukjoon Kwon, Youngchan Kim, Sung O. Park, Gwan Yeong Jung, Eunhye Shin, Wan-Gu Kim, Hyungdong Lee, Gyeong Hee Ryu, Minseok Choi, Tae Hyeong Kim, Junghoon Oh, Sungjin Park, Sang Kyu Kwak, Suk Wang Yoon, Doyoung Byun, Zonghoon Lee, Changgu Lee

**Affiliations:** 1 Mechanical Test & Analysis Section, Korea Electric Power Corporation Nuclear Fuel, Daedeok-daero 989 beon-gil, Youseong-gu, Daejeon 305-353, Korea; 2 School of Mechanical Engineering, Sungkyunkwan University, 2066 Seobu-ro, Jangan-gu, Suwon 440-746, Korea; 3 Department of Mechanical Engineering, The Ohio State University, 281 W. Lane Ave. Columbus, Ohio 43210, USA; 4 SKKU Advanced Institute of Nanotechnology, Sungkyunkwan University, 2066 Seobu-ro, Jangan-gu, Suwon 440-746, Korea; 5 School of Energy and Chemical Engineering, Ulsan National Institute of Science and Technology, Ulsan 689-798, Korea; 6 Department of Physics, Sungkyunkwan University, 2066 Seobu-ro, Jangan-gu, Suwon 440-746,Korea; 7 School of Materials Science and Engineering, Ulsan National Institute of Science and Technology, Ulsan 689-798, Korea; 8 New Material Team, Future Device R&D Department, LG Electronics Advanced Research Institute, 38 Baumoe-ro, Seocho-gu, Seoul 137-724, Korea; 9 Department of Chemistry and Chemical Engineering, Inha University, Incheon 402-751, Korea

## Abstract

The high-volume synthesis of two-dimensional (2D) materials in the form of platelets is desirable for various applications. While water is considered an ideal dispersion medium, due to its abundance and low cost, the hydrophobicity of platelet surfaces has prohibited its widespread use. Here we exfoliate 2D materials directly in pure water without using any chemicals or surfactants. In order to exfoliate and disperse the materials in water, we elevate the temperature of the sonication bath, and introduce energy via the dissipation of sonic waves. Storage stability greater than one month is achieved through the maintenance of high temperatures, and through atomic and molecular level simulations, we further discover that good solubility in water is maintained due to the presence of platelet surface charges as a result of edge functionalization or intrinsic polarity. Finally, we demonstrate inkjet printing on hard and flexible substrates as a potential application of water-dispersed 2D materials.

## Abstract (web summary)

The hydrophobic surfaces of many two-dimensional (2D) materials prevents direct exfoliation in water without the use of chemical, surfactant, or surface treatments. Here, the authors exfoliate and disperse 2D materials in pure water via simple control of the temperature of the sonication bath and storage.

## Graphical abstract

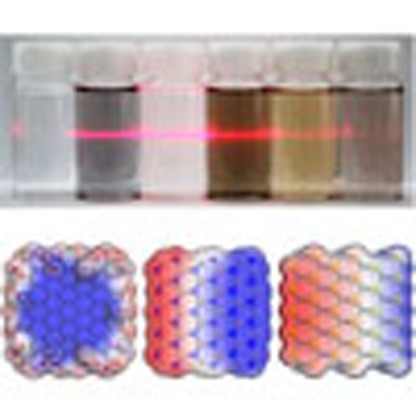

Two-dimensional (2D) sheets of graphene, hexagonal boron nitride (h-BN) and MoS_2_ have been studied vigorously in recent years because of their attractive mechanical, electrical, optical and chemical properties^1^,^2^,^3^,^4^,^5^,^6^. Although these materials all have atomic-level thicknesses, they have different physical properties, and so can complement each other in many ways. For example, because graphene is a conductor, h-BN an insulator, and MoS_2_ a semiconductor, they can be combined to fabricate field-effect transistors or electric circuits consisting purely of 2D materials^7^. In mechanical applications, they can be used as lubricating coatings or composites either with or without electric conductivity depending on their electrical properties. In numerous applications, such as batteries, surface coatings, composites, solar cells and catalysts, they need to be synthesized with platelet shapes in large quantities and most preferably at low cost. Dispersion in solvents, and particularly in cheap solvents, is the best approach to material preparation so as to satisfy these conditions. Since the surfaces of these materials are mostly hydrophobic^8^, it is difficult to exfoliate and dissolve them directly in water without the use of chemical, surfactant or surface treatments^9^,^10^.

In this paper, we present a simple method for the exfoliation of bulk-layered materials and the dispersion of the exfoliated 2D platelets in pure water, which are achieved by merely controlling the temperature of the sonication bath and storage. We tested this approach on several 2D materials, that is, graphene, h*-*BN, MoS_2_, WS_2_ and MoSe_2_, and observed that, in general, high temperatures favour both exfoliation and dispersion stability. However, the dispersion mechanisms of these materials vary because of their dissimilar surface and physical properties. We envision that such 2D material solutions can be produced in large volume at low cost, and that they are suitable for printing various patterns without much technical hindrance.

## Results

### Exfoliation and dispersion 2D materials in water

We used bulk-layered material powders to synthesize atomically thin platelets in water. The powders were added to deionized water and sonicated for ∼60 h in a bath sonicator (See Methods for materials and sample preparation). To control the bath temperature, we simply adjusted the cold water circulation valve in the bath so as to provide cooling or heating. When cold water circulation was stopped during sonication, the temperature increased to 60 °C within a few hours because of the dissipation of sonic energy. When cold water was circulated, the temperature was maintained at 30 °C. After sonication, the solutions of the exfoliated 2D materials were centrifuged at 600 relative centrifugal force to separate the thin flakes. The suspensions of thin 2D materials in water were stored at either a high temperature (60 °C) or a low temperature (20 °C) for 1 month to determine the temperature dependences of their long-term stabilities. In the synthesis and storage experiments, we used high (60 °C) and low (30 °C) temperatures for exfoliation and high (60 °C) and low (20 °C) temperatures for long-term solution stability studies of the materials.

**Figure 1:**
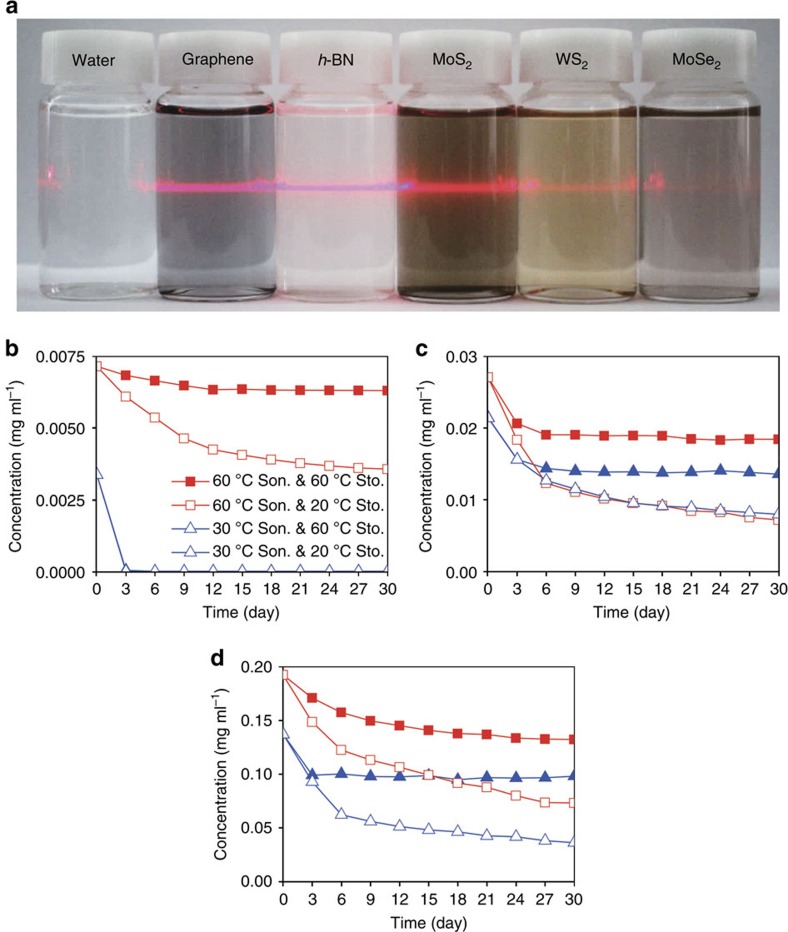
Temperature-dependent solution stabilities of the 2D materials in water. (**a**) Photographs of solutions of five 2D materials dispersed in deionized water for one month. The laser light across the solution bottles provides visual assistance because some suspended materials such as h*-*BN are not clearly visible. The long-term solution stabilities of (**b**) graphene, (**c**) h-BN and (**d**) MoS_2_ sonicated at the high (60 °C) and low (30 °C) temperature and stored at high (60 °C) and low (20 °C) temperatures. In (**b**–**d**), squares and triangles denote high and low temperature sonication and solid and blank denote high and low temperature storage, respectively. In (**b**), two types of triangles almost overlap because of fast precipitation.

Figure 1a shows various 2D materials dispersed in water; these dispersions were exfoliated and stored at a high temperature (60 °C). The nanoparticles were found to be suspended stably for longer than 1 month. The colours of the materials in water are as follows: black (graphene), milky-white (h*-*BN), dark-yellow (MoS_2_), yellow (WS_2_) and light-brown (MoSe_2_). All of the tested materials were found to be stably dispersed by high-temperature sonication and storage. By measuring the light absorption percentage over time with a ultraviolet–visible spectrophotometer, we determined the long-term stabilities of the dispersions of the 2D materials in water (See Methods for ultraviolet–visible measurements). These stability results indicate that the materials sonicated and stored at the high temperature remain stable for one month, as shown in Fig. 1b–d. After 10 days, most of the materials became stable, with little sedimentation afterwards. The graphene sample was found to exhibit the highest stability, with 90% of the suspension present even after 1 month. We observed that the colour of the graphene solution did not change noticeably even after 1 year. This high stability of our graphene sample is probably due to our optimization of the graphene synthesis process. The concentrations of dispersed flakes from high-temperature sonication and high temperature storage were 0.0065, 0.018 and 0.13 mg ml^−1^ for graphene, h-BN and MoS_2_ after one month respectively. Compared to the concentrations of 2D materials dissolved in organic solvents or surfactants, which are listed in Supplementary Table 1 (See Supplementary Table 2 for the sizes of exfoliated flakes), that of graphene is relatively low, but those of the other materials are comparable^10^,^11^,^12^,^13^,^14^,^15^. Since the dispersion condition was not optimized for high yield, the dissolution level of these nanoparticles is likely to be improved further as will be discussed later in concluding remarks.

Now, the materials exfoliated at the low temperature (30 °C) were found mostly to be satisfactorily suspended although the overall yield was lower than those sonicated at the higher temperature. However, only the graphene flakes precipitated quickly within three days, which indicates that low-temperature-sonicated graphene is unstable in water (Fig. 1b). To investigate the dependence of the dispersion stability on the storage temperature, the materials exfoliated at 60 and 30 °C were stored at either 60 or 20 °C. Overall, those stored at the high temperature were found to be more stable than those stored at the low temperature roughly by twice, as shown in Fig. 1b–d (Supplementary Fig. 1 for WS_2_ and MoSe_2_). According to the DLVO theory on colloid stability^16^ , nanoparticles in a solvent interact with each other through van der Waals force, which exerts the attractive and short-range force, and electrostatic force, which exerts the repulsive and long-range force. Between the two forces, the electrostatic force is important to make the suspension stable by preventing aggregation of the particles. Usually, electric double layer is formed around the nanoparticles by ions dissolved in the solvent, and it causes the particles to repulse each other. The thickness of the electric double layer depends on temperature, and increases with temperature. Hence, at higher temperature, the repulsive force also increases and the stability is supposed to be enhanced. Although stability seems to decrease over time in the case of low-temperature storage, we observed that precipitation ceases completely after ∼40 days and that the solutions maintain their same colour for longer than a year (Supplementary Fig. 2). Our exfoliation and solution stability results indicate that solutions of 2D materials that have been synthesized at the high temperature by sonication in water are quite stable at temperatures above room temperature.

### Characterization

**Figure 2:**
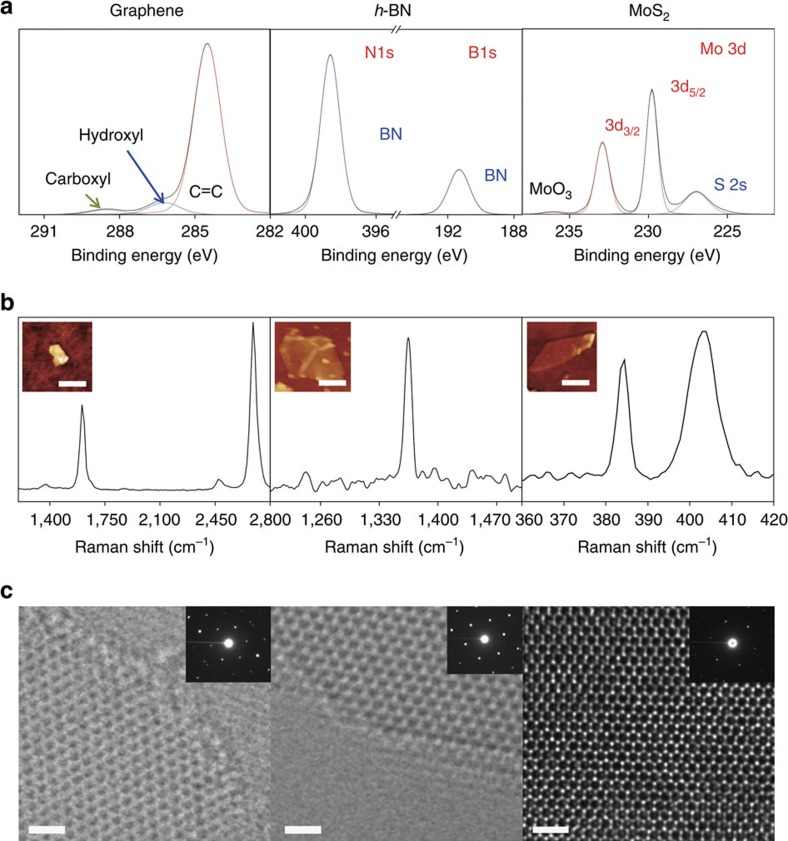
Analyses of the chemical compositions of the synthesized materials. (**a**) XPS, (**b**) Raman spectroscopy, and (**c**) high-resolution TEM. The scale bars in (**c**) are 1 nm.

**Figure 3:**
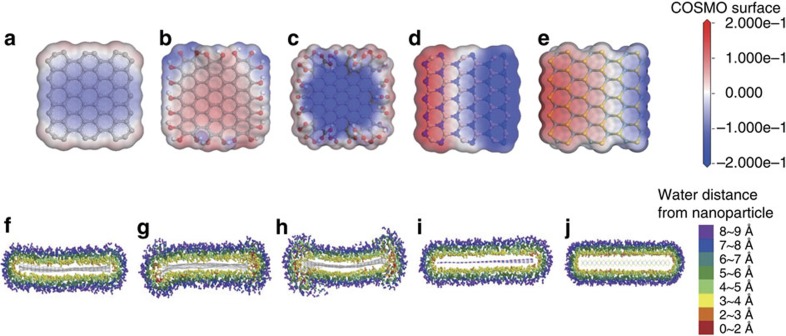
Molecular simulation results of solubility of 2D materials in water. Charge density of water around (**a**) pristine graphene, (**b**) –OH functionalized graphene, (**c**) –COOH functionalized graphene, (**d**) h-BN, and (**e**) MoS_2_. The isosurface is constructed from the COSMO-solvation model. The change of surface charge is much larger in polar nanoparticles. Water distributions around (**f**) pristine graphene, (**g**) –OH functionalized graphene, (**h**) –COOH functionalized graphene, (**i**) h-BN, and (**j**) MoS_2_.

Two questions arise from the observed synthesis temperature dependences of the 2D materials. The first question is why graphene synthesized at the high temperature is stable in water, whereas graphene prepared at the low temperature is not, in contrast to the other 2D materials. The second question is how the other materials are stably dispersed in water even at the low sonication and storage temperatures. Previous research into graphene dispersion might provide some insight. Although graphene does not readily dissolve in water, graphene oxide is well dispersed because of its surface functional groups^10^,^17^,^18^,^19^,^20^. Hence, it is possible that the graphene exfoliated at the high temperature is functionalized, whereas the graphene arising from low-temperature sonication is not. To investigate the functionalization of these materials, we used X-ray photoelectron spectroscopy (XPS) and Fourier transform infrared spectroscopy (FTIR). The XPS spectrum of graphene synthesized with high-temperature sonication shows that it is functionalized with carboxyl and hydroxyl groups (Fig. 2a), which can be identified from peaks at 288.6 eV (carboxyl group) and 286.2 eV (hydroxyl group). In contrast, the spectrum of graphene prepared with low-temperature sonication indicates that only a negligible number of such functional groups are present (Supplementary Fig. 3a). FTIR spectra of graphene samples synthesized with high- and low-temperature sonication are displayed in Fig. 3. That of the graphene sample from high-temperature sonication shows appearance of new peaks at 1,024 and 1,094 cm^−1^, corresponding to C–O stretches in hydroxyl groups, and at 1,260 cm^−1^, corresponding to a C–O stretch in carboxyl or epoxy group, while they were not observed in the FTIR spectra of graphite and graphene samples from low-temperature sonication^21^. It clearly confirms that high-temperature sonication leads to successful functionalization of oxygen-containing hydrophilic groups on graphene network. In contrast, there is no evidence of functionalization in the XPS and FTIR data for h-BN and MoS_2_ (Fig. 2a and Supplementary Fig. 4).

To determine whether these functional groups of graphene are present on the surface, as in graphene oxide, or on the edges, we performed Raman spectroscopy on a single-layer graphene flake obtained with high-temperature sonication. The intensity of the D-peak in the Raman spectrum of the central area is negligible, which indicates the bare existence of functionalization or defects (Fig. 2b). The peaks in the Raman spectrum of bulk graphite, which is the raw material used in the synthesis of the graphene sample, have similar shapes, except for that of the D-peak; this difference is due to the layer edges (Supplementary Fig. 5a). The Raman spectra of the other materials indicate that they are not damaged structurally by the sonication process (Fig. 2b and Supplementary Fig. 5b,c). Also they prove that the materials were exfoliated down to monolayer from high 2D/G peak ratio in graphene spectrum^22^, which is the clear evidence of single-layer graphene, and the gap of 18.5 cm^−1^ between E_2g_ (ref. 1) peak and A_1g_ peak in MoS_2_ spectrum, which is also a solid proof of single-layer MoS_2_ (ref. 23). We also could confirm the exfoliation of monolayer MoS_2_ through photoluminescence (PL) spectroscopy, since monolayer MoS_2_ has distinct peak positions and high intensity at around 1.82 and 1.97 eV compared with thicker layers^24^. (See Supplementary Fig. 6) For h-BN, we could hardly identify single-layer due to the small size and poor optical contrast of the flakes (in average 200 nm)^25^, thus we identified samples of a few layers optically and got the Raman data. However, thinner layers such as monolayer could be identified through transmission electron microscopy (TEM) as explained below.

To assess the quality and distribution of thickness of the exfoliated flakes, we collected high-resolution TEM images, as shown in Fig. 2c. The in-plane crystalline structures of the materials are well ordered without defects. These images reveal that sonication results in little damage to or chemical reaction on the surfaces of these materials. On the graphene edge, the atoms are not discernible, which is an indirect indication of edge functionalization. In contrast, the edge atoms of h*-*BN are well ordered without signs of functionalization. A previous study of the water dispersion of h*-*BN speculated that good dispersion is due to edge functionalization despite its hydrophobic surface^25^. However, our TEM observations show that h*-*BN does not dissolve because of functionalization. In addition, a previous theoretical study provides evidence that h*-*BN is hardly oxidized due to the thermodynamic instability of its oxide^26^. The TEM image of MoS_2_ also shows that its crystal structure is not damaged or oxidized by sonication, at least in the centre. Our TEM observations also confirm the results obtained with optical spectroscopies for the structures and functionalization of graphene, h*-*BN, and MoS_2_. We also determined the thickness distributions of the suspended flakes by using low-resolution TEM imaging, as shown in Supplementary Fig. 7. Overall, the dispersed materials consist mostly of 2–3 layers and the portion of monolayer was 10–20 %. The flake size is ∼200–300 nm.

Our spectroscopy and TEM results indicate that graphene sonicated at the high temperature is functionalized only on its edges and that these functional groups enhance the solubility of graphene in water. This conclusion raises another question: how is graphene functionalized when sonicated at the high temperature. In an ultrasonic bath, cavitation occurs because of abrupt pressure fluctuations. Cavitation is a phenomenon in which bubbles are formed in water and collapse within milliseconds. Bubble collapse induces a quite high local temperature of ∼5,000° C and a high local pressure of tens of thousands of bars^27^. Water can decompose into chemically volatile H^+^ and OH^−^ ions at such conditions, so cavitation can provide sufficient energy to break bulk-layered materials and induce the chemical reactions of such materials with water. This type of sonochemical reaction (or sonochemistry) has been used in the synthesis of diverse nanomaterials^28^. The graphene edge has dangling carbon atoms, so more easily reacts with radicals or ions than does the in-plane area^29^. In cavitation by ultrasonic agitation, usually the sonic pressure amplitude should be greater than the difference between the atmospheric pressure and the boiling pressure at a given temperature; this difference is ∼ 1 atm under ambient conditions. When sound propagates in matter, the pressure is attenuated by energy absorption. In water, the attenuation level is higher at lower temperatures, which means that the sonic pressure is lower at lower temperatures because cold water can absorb the heat dissipated from the sonic energy easily^30^,^31^.We measured the sonic pressure in the bath at two different temperatures: 60 °C and 30 °C. The sonic pressure at 60 °C was found to fluctuate by as much as 2.4 atm, which can easily induce cavitation, whereas at 30 °C the sonic pressure fluctuates by as much as 0.46 atm (Supplementary Fig. 8). These temperature-dependent sonic pressure measurements suggest that the threshold temperature for the active sonochemical functionalization of graphene would be around 40 °C, above which graphene can be dispersed with long-term stability.

For the 2D materials other than graphene, other factors must be present that induce their dissolution in water regardless of the synthesis temperature. In contrast to graphene, h*-*BN, MoS_2_, WS_2_ and MoSe_2_ dispersions remain stable when dispersed via high- and low-temperature sonication, which indicates that edge functionalization is not the cause of the high water solubility of these materials. These materials are electrically less conductive than graphene, so an electrical double layer can form on their surface and thus the materials can be readily suspended in water without agglomeration or sedimentation. This hypothesis can be assessed by performing zeta-potential measurements because nanoparticle solution stability is closely related to the electrical double layers on nanoparticle surfaces. In general, dispersed materials with zeta-potential values near or <−30 mV are stable^32^,^33^,^34^. The zeta-potential values of the h*-*BN, MoS_2_, WS_2_ and MoSe_2_ dispersion samples are all ∼ −30 mV, irrespective of the sonication temperature (Supplementary Fig. 9). The zeta-potential of the high-temperature-sonicated graphene sample is similar. However, the zeta-potential of the graphene sample sonicated at the low temperature is −13 mV and this sample exhibits low solution stability. Therefore, the zeta-potential results support our argument for a temperature-dependent dispersion mechanism in water.

The pH of a dispersion solution can change during sonication because of sonochemical reactions, and this change might affect solubility. In the case of graphene, h*-*BN, and MoSe_2_, the pH varies little; hence, the pH does not appear to influence their solubility. However, the MoS_2_ and WS_2_ solutions became acidic after sonication (Supplementary Fig. 10). To investigate the effects of pH on solubility, we measured the pH of aqueous solutions of the raw material powders and found that they are similar to the pH values of the dispersed 2D material solutions. However, the particles in powder solutions mostly precipitate regardless of temperature, in contrast to the sonicated solutions; these results indicate that pH is not a major contributor to the high solubility of the 2D materials. To show that the 2D materials are dispersed in water due to exfoliation, we prepared control samples of graphite, which were just stirred at 60 and 30 °C and compared with the sonicated samples as shown in Supplementary Fig. 11. The simply stirred graphite particles of both temperatures precipitated quickly within several hours and faster than sonicated samples.

### Numerical calculation of water-nanoparticle interaction

In order to further investigate the solvation states of these materials, we performed molecular dynamics (MD) simulations and *ab initio* calculations for pristine graphene, functionalized graphene (hydroxyl (−OH) and carboxyl (−COOH) groups), h-BN and MoS_2_ (See Methods for calculation methods). Hydroxyl- and carboxyl-functional groups on graphene materials are chosen as possible functionalities from above XPS and FTIR characterization. Pristine graphene has a weakly positive charge due to dangling carbon atoms, and carboxyl-functionalized graphene has a stronger positive charge due to the electron-withdrawing carboxyl groups, whereas the electron-donating hydroxyl groups render graphene flakes negatively charged. On the other hand, h-BN has an alternating charge distribution and exhibits strong polarity across the boron and nitrogen termination edges and MoS_2_ shows the negative charge on the surface by S atoms and counter charges inside by Mo atoms, which exhibit moderate polarity (Supplementary Fig. 12). Due to the surface charges of the materials, water molecules, which are polar, are attracted and cause charge distributions in water as shown in Fig. 3a–e. The numerical calculation results of interaction between the 2D materials and water show that the solvation level is highest for carboxyl-functionalized graphene, the intermediate for hydroxyl-functionalized graphene, h-BN and MoS_2_, and the lowest for pristine graphene (See Methods for calculation results). Furthermore, we investigated the distribution of water molecules around the nanoparticles (Fig. 3f–j and Supplementary Fig. 13). Water molecules within ∼ 6∼8 Å of the nanoparticles were found to reorient in charged layers so as to neutralize the charged nanoparticles, which means that edge effects due to functional groups or B and N, or Mo and S are dominant. This particular orientation of the water molecules leads to stronger interactions with the nanoparticles; the strengths of these interactions are estimated to be greater than that of the water–water interaction. Note that the overall attractive interaction energy is due to weak van der Waals interactions and strong electrostatic interactions. It has been found that a charge distribution or charge polarity on the surfaces of materials enhances their interactions with water molecules, hence functionalized graphene, h-BN and MoS_2_ are more water-soluble and stable than pristine graphene, which is in good agreement with our experimental observations.

### Printing application of the solutions

**Figure 4:**
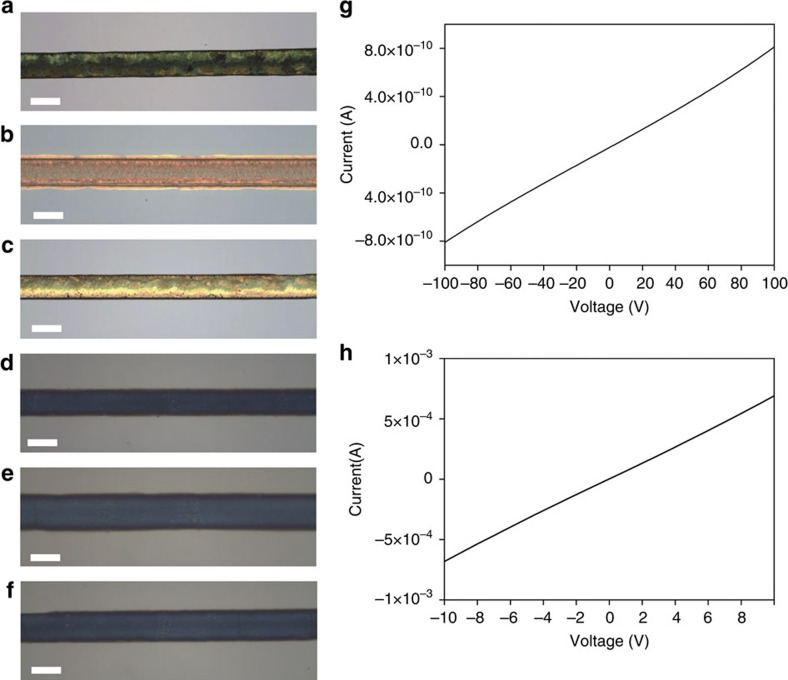
Inkjet printing results with 2D material solutions. Lines printed with pure-water inks of (**a**) graphene, (**b**) h*-*BN, and (**c**) MoS_2_, and mixed PEO-water inks of (**d**) graphene, (**e**) h-BN, and (**f**) MoS_2_. Electrical conductivity measurements for (**g**) a pure-graphene electrode and (**h**) a mixed PEO-water graphene electrode. The scale bars in (**a**–**c**) and (**d**–**f**) are 300 and 20 μm.

Our water-based synthesis method is facile and will be potentially cost-effective, and the materials are well dispersed; thus, diverse applications should be possible. We performed inkjet printing with the dispersed 2D material solutions since the as-prepared solutions can be used directly as inks. First, we used the nozzle printing method applying a hydraulic pressure to the nozzle to draw lines on Si and Si/SiO_2_ with graphene, h*-*BN, and MoS_2_ dissolved in pure water as shown in Fig. 4a–c (See Methods for printing methods). Since the viscosity of water is quite low, printing with inks based on pure water is extremely challenging. We increased the concentration of the 2D materials in the solution by evaporating major portion of water and controlled the substrate temperature at 50 °C to achieve successful printing with water solutions. There is an intrinsic limitation on the reduction in line width that can be achieved with water-based inks due to the low viscosity of water, so we mixed a polymer poly (ethylene oxide) (PEO) with the water solutions of the 2D materials to increase their viscosity and performed electrohydrodynamic (EHD) printing by applying an electric field between the nozzle and the substrate, as shown in Fig. 4d–f (see Methods for printing details). With this approach, we were able to draw lines with a width of 20 μm, which can be further reduced by replacing the nozzle with one with a smaller diameter and optimization. We could also print other patterns such as letters and mesh on the hard substrates of SiO2/Si and Si and, and a mesh pattern on a flexible substrate as shown in Supplementary Fig. 14.

Graphene is a prominent conductor, so we characterized the electrical properties of the graphene printed lines and films as shown in Fig. 4g,h and Supplementary Fig. 15. The PEO-graphene composite was found to exhibit a conductivity of 6 × 10^−5^ S m^−1^, which is comparable to that of a previous graphene-contained composite^35^. Unlike the graphene composite, in which the conductive graphene flakes are separated by polymer, the flakes are directly connected with each other in a pure-graphene electrode, hence the conductivity was much higher, with a value of 50 S m^−1^. Since the graphene flakes would be poorly stacked due to the rapid printing and drying process in this experiment, the electrical property appears relatively low compared with graphene or reduced graphene oxide samples synthesized by other methods^11^,^12^,^36^,^37^. Hence, we fabricated graphene films by filtering the solution through an anodisc filter and found the electrical conductivity to be 440 S m^−1^, which is about one order higher than the printed film (See methods and Supplementary Fig. 15). Because this film was made by a slower process, the stacking would be more ordered. Therefore, we judge that there is a room for further enhancement of electrical properties by improving the film fabrication process. This level of conductivity is quite encouraging that the graphene films would be able to be used as an electrode for electric devices. The printing results indicate that the water-dispersed 2D material inks are promising for printed electronics, with no significant hindrances to practical applications.

## Discussion

The results of our dispersion experiments with the 2D materials show that sonication at a high temperature results in the synthesis of 2D materials with good dispersion stabilities in water. This ability to use pure water to exfoliate and disperse 2D materials will enable not only cost-effective industrial and commercial applications but also facilitate research that requires water-based experiments. The heat required to raise the sonication bath temperature originates from the dissipation of sonic energy, so no additional heat energy is required for the production of the exfoliated materials. To date, ultrasonic cavitation has only been used for the physical exfoliation of 2D materials via the breaking of bulk lamellar materials into thinner flakes^15^. However, in this study, we demonstrated the first synthesis of water-soluble graphene by performing a sonochemical reaction through temperature-controlled sonication. Since cavitation is the key factor to the 2D materials dispersion in water, the particle dissolution yield can be improved by heating the water more and lowering the pressure, which enhances the level of cavitation. Our results and findings further the development of large-volume and economical synthesis methods for 2D materials using sonochemistry. We have achieved inkjet printing with water-dispersed 2D material inks, which thus have significant potential for real-life applications.

## Methods

### Materials and sample preparation

For sample preparation, we purchased graphite and MoSe_2_ powders from Alfa Aesar. h-BN powder was purchased from Momentive. MoS_2_ and WS_2_ were purchased from Sigma Aldrich.

To exfoliate and disperse 2D materials in water from the source powders, we used a bath sonicator, manufactured by Onejon Ultrasonic in Korea. Each material (20 mg) was sonicated in deionized water (200 ml) for 60 h. The operation power was 20 W and frequency was 40 kHz. In addition, the cooling water circulation equipment was built in the sonicator to control the sonication bath temperature. The resulting solution was centrifuged at 600 relative centrifugal force for 30 min using a high speed centrifuge (Supra 25K, HANIL SCIENCE INDUSTRIAL). The final solution was kept either in an atmospheric condition (20 °C) or in an oven (60 °C).

### Dispersion stability measurement

We first measured the concentration of the 2D materials right after sonication and centrifugation by weighing the mass of the dried flakes. When measuring the mass, we prepare two identical solution samples, so that one can be used for mass measurement, and the other one for concentration change measurement. We observed the concentration change (or stability) of the dispersions using the ultraviolet–VIS–NIR spectrophotometer (UV-3600, SHIMADZU) to confirm how long the flakes are stably suspended in water. We put the solutions in standard 1-cm path quartz cell and measured the absorbance for 1 month with the interval of 3 days. The stability represents the absorbance at 650 nm normalized by the first measured value. The absorbance at 650 nm has been normally used to compare the stability of graphene in a dispersion study^15^,^38^,^39^. The stability of MoSe_2_ and WS_2_ in water is shown in Supplementary Fig. 2 (See the main paper for the stability of graphene, h-BN and MoS_2_). The WS_2_ stored at high temperature is considerably stable in water for 1 month while the stability at low temperature a little bit lower over the time. We can know the stability of WS_2_ suspended in water is dependent on the storage temperature, like graphene, h-BN and MoS_2_ dispersions. However, the solubility of MoSe_2_ dispersed in water is high at both high and low storage temperature. It is worth noting that once MoSe_2_ is exfoliated in water, the dispersion can be very stable regardless of the storage temperature.

### XPS measurement

XPS analysis was performed to investigate the functional groups of graphene, h-BN and MoS_2_ flakes exfoliated at each temperature. Each suspension of graphene, h*-*BN and MoS_2_ was dropped onto a quartz substrate using a pipette until flakes totally covered the substrate. The thin films on the quartz substrate were annealed at 160 °C under Ar gas flow to dry up the water moisture. We analysed the samples using K-Alpha X-ray Photoelectron Spectrometer system with monochromated Al X-ray sources. The survey scan was −50 eV, and the step size was 0.1 eV. Flood gun was used for charge compensation.

Supplementary Fig. 3a shows the XPS data of graphene sonicated at low temperature. The spectrum does not have functional groups, which are shown in high-temperature sonicated graphene. Supplementary Fig. 3b shows the XPS data of the MoS_2_ powder. There are some peaks representing MoS_2_, such as 3d3/2 and 3d5/2 peaks, related to Mo and S 2s peak due to S bonded to Mo. MoO_3_ peak also exists, and this peak means the oxide bonded to Mo. There is almost no difference between the MoS_2_ flakes and MoS_2_ powder. It is noted that the MoO_3_ peak is observed at both spectra, and the atomic per cent and ratio of MoS_2_ flakes are similar to those of the MoS_2_ powder. From the MoO_3_ peak and the atomic per cent of O, we can conclude that the MoS_2_ flakes in water were not functionalized, as opposed to graphene (see the main manuscript for the graphene). Also, the h-BN flakes were not functionalized during sonication process, as shown in Supplementary Fig. 3c.

### FTIR spectroscopy

FIIR analysis was performed to investigate functional groups of graphene h*-*BN, and MoS_2_ flakes exfoliated at each temperature. Each suspension was filtered through an anodisc membrane by using an aspirator, and then the filtered film was transferred to a silicon substrate. The thin film on the substrate was annealed at 160 °C under Ar gas flow to dry up the water moisture. We analysed the h-BN and MoS_2_ samples using Bruker IFS-66/S Fourier Transform Infrared Spectroscopy system. FTIR spectra of graphene and graphite samples were obtained from KBr pellets using an FTIR vacuum spectrometer (Bruker VERTEX 80V, Bruker, Germany) with 64 scans.

### Raman spectroscopy

We performed the Raman spectroscopy to confirm the quality of the nanosheets of graphene, h-BN, MoS_2_ exfoliated in water and to compare with the raw materials. Films on silicon oxide substrate for the measurement were prepared as the films for FTIR spectroscopy except for the substrate material. The excitation wavelength was 532 nm. Supplementary Fig. 4 Raman shows the spectra of the powders of raw materials, which are similar to the spectra of the water-exfoliated flakes. From Raman spectra of the four materials, we could not find oxide peaks, which reveals that they were not oxidized during the synthesis 40,41,42.

### PL spectroscopy

We performed the photoluminescence analysis to confirm the exfoliation of MoS_2_ to single and few layers. The MoS_2_ flakes were deposited on a silicon substrate. The excitation wavelength was 532 nm. The strong intensity of peaks at 1.82 eV (680 nm) and 1.97 eV (630 nm) confirms the existence of monolayers among the flakes, and those peaks are contrasted with bilayer and trilayer flakes shown in Supplementary Fig. 5 (ref. 43).

### Low-resolution TEM image and distribution of sheet thickness

The nanosheets were further analysed using a TEM. A drop of dispersion was casted on holy carbon grid (400 mesh) for the measurement. Low-resolution TEM imaging was conducted using FE-TEM manufactured by JEOL Ltd. with the acceleration voltage of 200 kV. The upper row of Supplementary Fig. 5 shows the typical images of 2D materials. We can observe monolayer, folded mono- or bi- layer, and few layers. In addition, thickness distributions of sheets were plotted through the investigation in TEM results as shown in the lower row of Supplementary Fig. 5. Overall, about 10% of the flakes were monolayer, and about 87 % of observed flakes were thinner than 5 layers. In fact, we can know that the dispersions prepared by present method contain very thin nanosheets.

### Atomic resolution TEM imaging

Specimens were analyzed using an aberration-corrected FEI Titan Cubed TEM (FEI Titan^3^ G2 60–300), which was operated at 80 kV acceleration voltage with a monochromator.

### Measurement of the sonic pressure

The beaker was placed in the centre of the bath sonicator. The beaker was filled with deionized water without particles in order to prevent contamination of the probe and the artifacts from the particles. The sonic wave was measured at the centre of the beaker for 5 ms with a hydrophone (Gearing & Watson D/140) to obtain sonic pressure data.

Supplementary Fig. 6 shows temporal waveforms and spectra for the two different temperatures of the sonication bath. The main frequency peak appears at around 37 kHz. The frequency peaks can vary depending on the temperature and the dissolved particles. The measured sonic pressure values at high (60 °C) and low (30 °C) temperatures are 237,000 Pa and 46,000 Pa.

### Electrokinetic potential of 2D flakes in water

We measured zeta potential of the centrifuged dispersions using zetasizer (NANO ZS, Malvern instruments). Usually, when the zeta potential is below −30 mV, the dispersion quality of flakes in water is quite excellent^10^. The graphene sonicated at high temperature was below–30 mV. Just considering the zeta potential value, graphene synthesized by our method appears better compared with a reduced graphene oxide^10^. However, the graphene prepared at low temperature has poor zeta potential. For the other materials, the zeta potential was, in general, lower with high temperature sonication.

### pH measurement

The hydrogen exponent of the aqueous solutions was measured to confirm the state of the solution using pH metre (E-sweep, Seiko Instruments Inc.). Supplementary Fig. 10 shows the pH values of all materials dispersed in water.

### Molecular modelling and simulation

We constructed five platelet-nanoparticle models, which are pristine graphene, hydroxyl-functionalized (–OH) graphene and carboxyl-functionalized (–COOH) graphene with basal planes of the side length about 5 nm, h-BN, and MoS_2_ with the edge types of Mo-edge with 0% S atoms and S-edge with 100% S atoms. Note that we fully functionalized edges of graphene with functional group for each simulation to observe clearer effects. Mulliken-based partial charges of the models were estimated by the population analysis method incorporated in DMol^3^ (refs 44, 45), which is able to perform the density function theory (DFT) calculation, through investigating nanoribbon models of pristine graphene, functionalized graphenes, h-BN, and MoS_2_. Note that partial charges of hydrogen (+0.41e) and oxygen (−0.82e) in water molecule were used. Supplementary Fig. 12 shows the results of charge distributions of the nanoparticles. For investigating charge distribution of water molecules surrounding nanoparticles, we have performed MD simulations of each nanoparticle submerged in water with a box size of 10 × 10 × 3.6 nm^3^. We used different potential parameters for each material to describe inter- and intra-molecular interaction. The COMPASS forcefield for pristine and functionalized graphenes, DREIDING forcefield for h-BN and fitted forcefield originated from works by Varshney *et al.*^46^ and Morita *et al.*^47^ for MoS_2_ were used, respectively (Supplementary Table 3). MD simulations have been performed with the isothermal-isobaric ensemble (that is, NPT, where N is the number of molecules, P is pressure, and T is temperature) for 50 ps with a time step of 1 fs followed by the canonical ensemble (that is, NVT, V is volume) for over 200 ps with the same time step at 1 atm and 60 °C. For monitoring temperature and pressure, Berendsen thermostat and barostat were used, respectively. For the detailed analysis, we partitioned the volume by isosurface shell contouring the equilibrated nanoparticle in 3D at an interval of 0.5 Å and averaged charges of hydrogen and oxygen atoms over the shell volume. In the same context, the non-bonding interaction energy (that is, van der Waals and electrostatic energy) between water molecules in each shell and nanoparticle has been calculated. The results are shown in Supplementary Fig. 13.

The solvation energy is calculated by the interaction of nanoparticles with implicit water molecules. As shown from Supplementary Fig. 12, we obtained the gas phase geometries of nanoparticles by the DFT calculation using DMol^3^ (refs 44, 45) with the generalized gradient approximation with the Perdew–Burke–Ernzerhof functional^48^. The effect of implicit water environment is included by using the COSMO(Conductor-like screening model)^49^ scheme. Unrestricted spin-polarized calculations were performed with basis set of DNP 4.4 level, and the SCF convergence criterion was set to be <1.0 × 10^−6^ ha. The calculated solvation energies for graphene, graphene with hydroxyl group, graphene with carboxyl group, h-BN, and MoS_2_ are −23.45, −66.46, −213.4, −129.6 and −130.3 kcal mol^−1^, respectively.

The Flory–Huggins parameter *χ* measures the interaction related to mixing of solvent and solute. As *χ* decreases, it can be considered that solute is more soluble in solvent. To obtain *χ*, the solubility parameter *δ* of nanoparticle has been estimated from MD simulation, of which the system contains 200 nanoparticles. We chose to use the Hansen solubility parameter, which is known to be good for predicting miscibility of polar molecules. The NPT MD simulation has been performed for the system to be at an equilibrated density at 298 K and subsequently, the NVT MD simulation has been performed for further equilibration. Dispersion and electrostatic solubility parameters were obtained from resulting configurations of the NVT MD simulation. Since the Hansen solubility parameter is composed of dispersion *δ*_d_, polar *δ*_p_ and hydrogen bonding *δ*_hb_ terms by the following relation^50^,


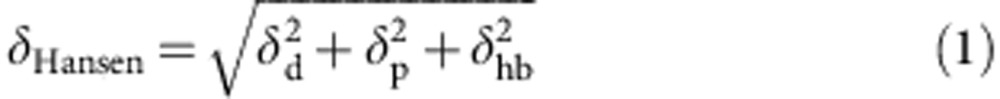


the three solubility parameters have been obtained with the help from the DFT calculation by the COSMO scheme. In particular, for the polar solubility parameter, the following equation was applied^51^


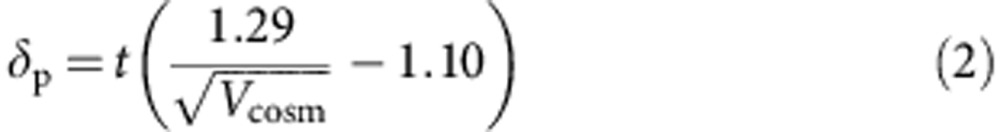


where *t* is dipole moment in the unit of Debye and *V*_cosm_ is COSMO volume(nm^3^). From these solubility parameters, the Flory–Huggins parameter was finally calculated by the following equation^50^


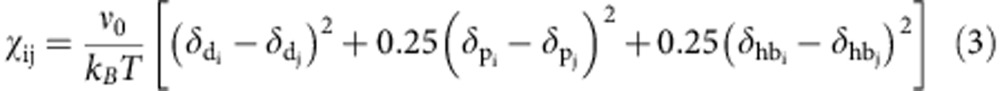


where the subscripts *i* and *j* represent each nanoparticle and water, respectively, and *v*_0_ is the solvent molecular volume, which is 30 Å^3^ from water.

The calculated Flory–Huggins parameters for graphene, graphene with hydroxyl group, graphene with carboxyl group, h-BN and MoS_2_ are 4.81, 3.05, 1.82, 4.45 and 4.26.

### Inkjet printing method

Two types of 2D material inks were used for inkjet printing: inks of pure-water solvent and inks of PEO and water mixture. For the PEO-mixed inks, 4 wt.% of PEO powder (average Mw∼1,000,000, Sigma Aldrich) was dissolved in a mixed solvent of 60 wt.% deionized water and 40 wt.% ethanol. Solutions of PEO in water/ethanol mixture were added to the graphene, MoS_2_ and h*-*BN suspension with proper ratio using a THINKY mixing machine (Thinky Inc, ARE-310) to make viscoelastic homogeneous inks.

We used the EHD printing machine (Enjet Inc, Korea) in this experiment. A micro-syringe pump was used to supply the 2D materials (graphene, MoS_2_ and h*-*BN)/PEO composite suspensions from a 1 ml syringe into the metallic nozzle. The nozzle was a 32G (inner diameter (I.D.): ∼0.23 mm, outer diameter (O.D.): ∼0.10 mm) stainless steel needle. The tip-to-the-collector distance was adjusted at around 2.5 mm to stabilize the near-field fibre jet to ensure the ohmic flow region. For EHD printing on Si and Si/SiO_2_ substrates, we put them on the metallic movable stage and applied high voltage around ∼1.8–2.2 kV. Although the Si/SiO_2_ substrate has an insulating surface, the inks can be successfully printed by the EHD jet printer, because the insulating layer is thin enough to maintain a high electric field between the nozzle tip and the grounded stage. With the EHD inkjet printing, complicated patterns like letters and meshes could be drawn as shown in Supplementary Fig. 14a-c. To draw a pattern with a pure water inks or on PET substrate, electric field cannot be applied since water is too conductive and PET is insulating. Hence, the ink was pushed merely by the syringe pressure. This method is called nozzle inkjet printing. The printed mesh pattern is shown in Supplementary Fig. 14d.

## Additional information

**How to cite this article**: Kim, J. *et al.* Direct exfoliation and dispersion of two-dimensional materials in pure water via temperature control. *Nat. Commun.* 6:8294 doi: 10.1038/ncomms9294 (2015).

## Supplementary Material

Supplementary Information Supplementary Figures 1-15, Supplementary Tables 1-3 and Supplementary References
