# Supplementary material for: Direct exfoliation and dispersion of two-dimensional materials in pure water via temperature control
Source: Nat Commun. 2015 Sep 15;6:8294. doi: 10.1038/ncomms9294 (PMC4579837; doi:10.1038/ncomms9294)
Supplement: Supplementary Information — Supplementary Figures 1-15, Supplementary Tables 1-3 and Supplementary References [file ncomms9294-s1.pdf]

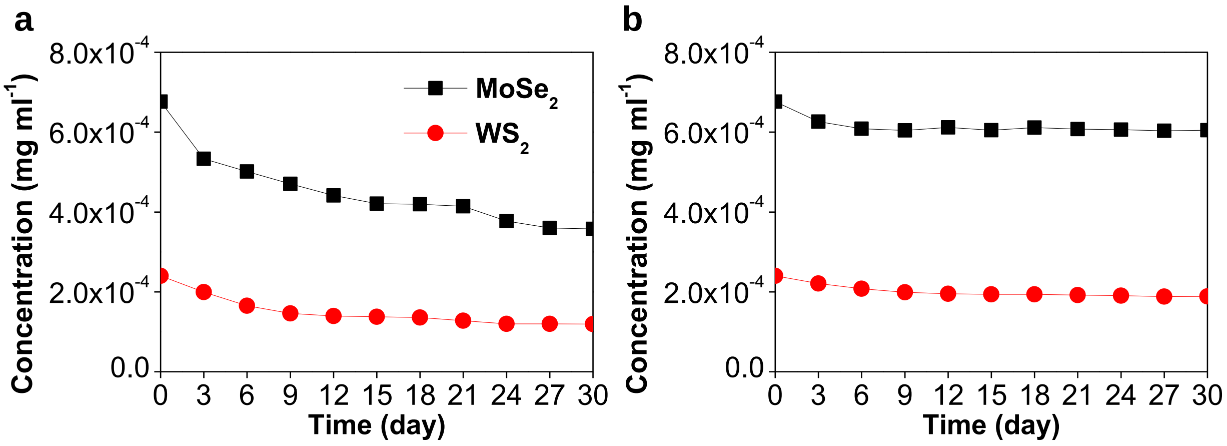

Supplementary Figure 1 Temperature dependent solution stability. (**a**) high temperature sonication and high temperature storage and (**b**) high temperature sonication and low temperature storage **of $MoSe_2$ (Black square) and WS_2 (red circle).**.

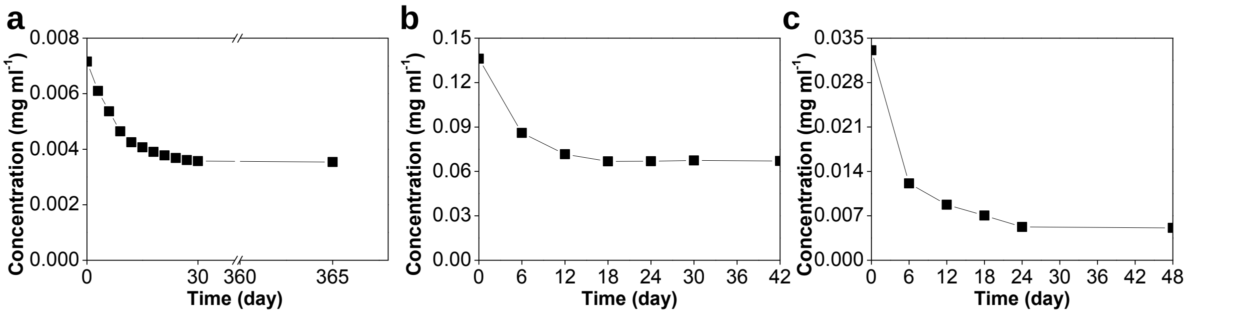

Supplementary Figure 2 Long term dispersion stability. (**a**) graphene, (**b**) h-BN, and (**c**) MoS2 samples of high temperature sonication and low temperature storage.

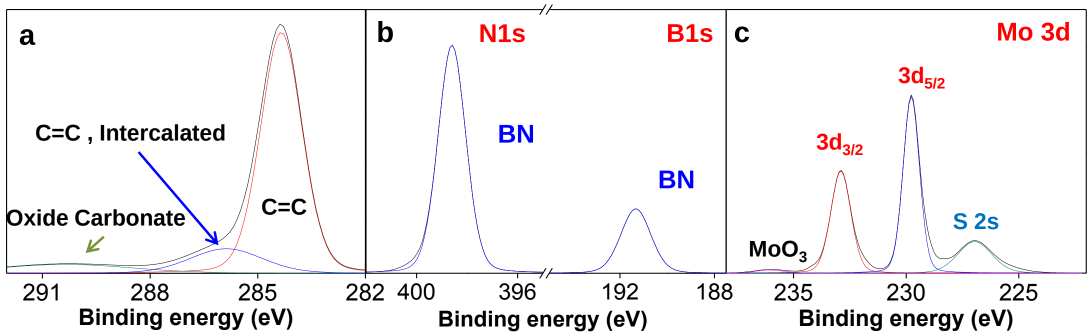

Supplementary Figure 3 XPS data. (**a**) graphene sonicated at low temperature, (**b**) *h*-BN powder, and (**c**) MoS_2 powder

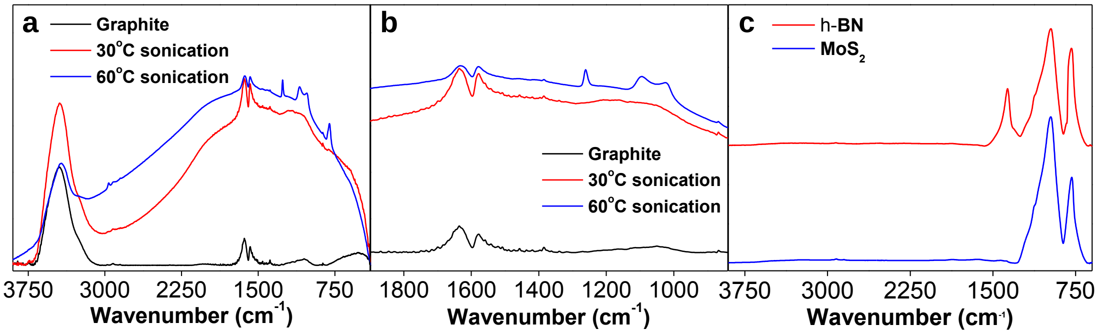

Supplementary Figure 4 FTIR spectra from exfoliated flakes. (**a**) high- and low-temperature sonicated graphene samples and a graphite sample at full region, (**b**) and at selected region, and (**c**) h-BN, and MoS2 from high- temperature sonication. Two peaks at 1,580 and 1632 cm^{-1}, which correspond to C=C stretches, are observed in all spectra.[1] A peak at 1,260 cm^{-1} corresponds to a C-O stretch in carboxyl or epoxy groups.[2]

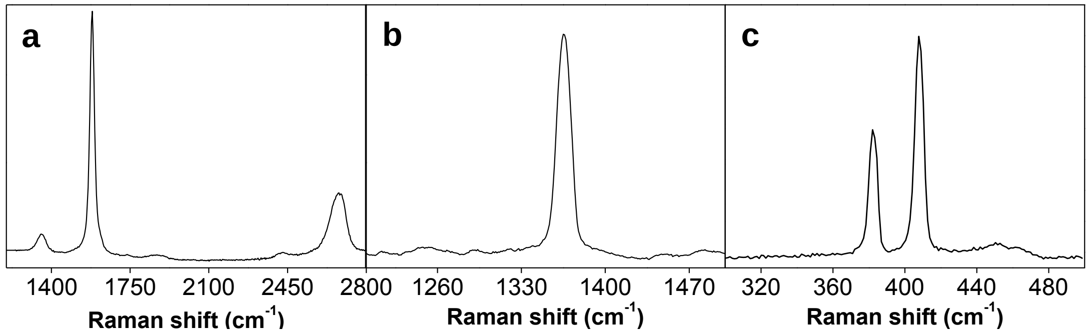

Supplementary Figure 5 Raman spectra for raw materials. (**a**) graphite, (**b**) *h*-BN, and (**c**) MoS_2

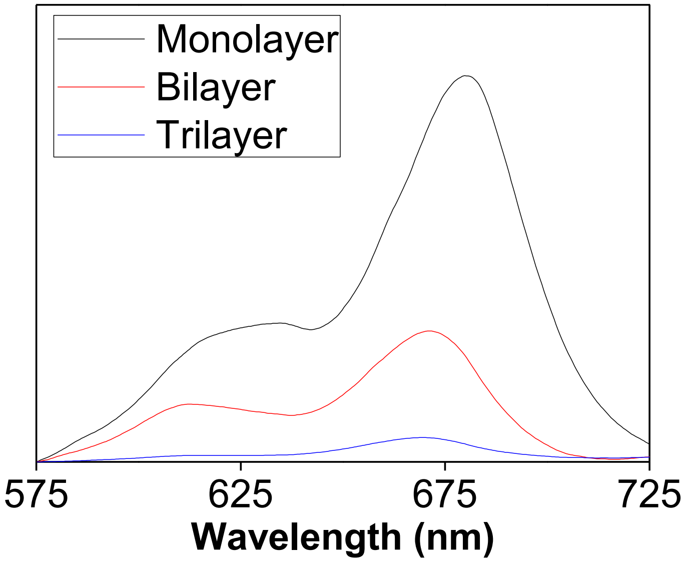

Supplementary Figure 6 Photoluminescence spectrum of exfoliated mono-, bi-, and tri-layer.

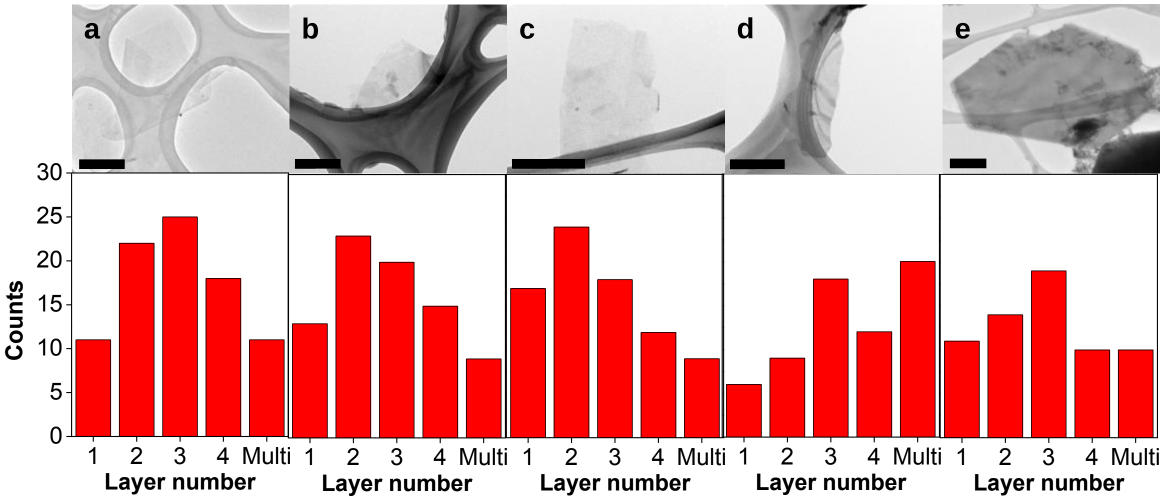

Supplementary Figure 7 TEM images (top row) and thickness distribution (bottom row) of exfoliated flakes. (**a**) graphene, (**b**) *h*-BN, (**c**) MoS_2, (**d**) WS_2, and (**e**) $MoSe_2$. The scale bars are 100nm.

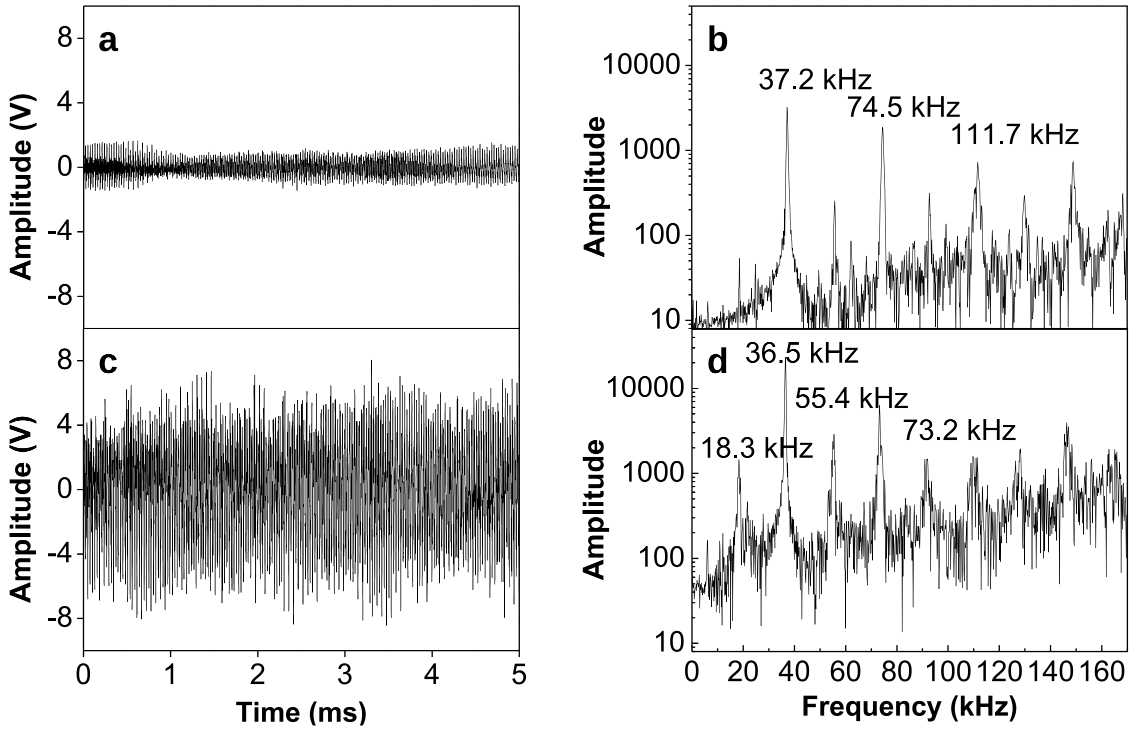

Supplementary Figure 8 Temporal waveforms and frequency spectra of sound pressure field. (**a**) Temporal waveform at 30°C (**b**) frequency spectrum at 30°C (**c**) temporal waveform at 60°C (**d**) frequency spectrum at 60°C

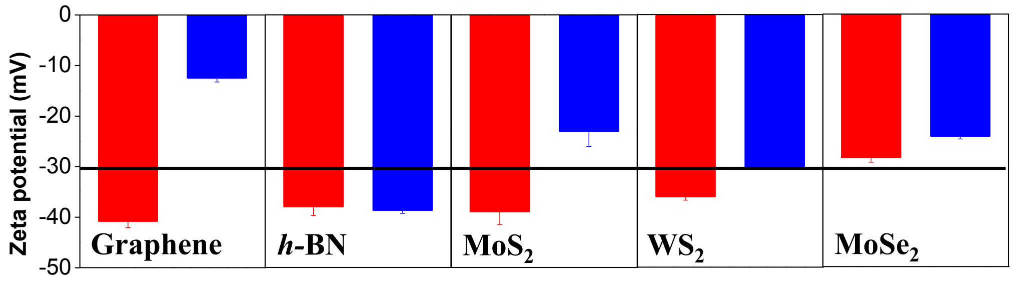

Supplementary Figure 9 Zeta potential measurement for high (red) and low (blue) temperature sonicated 2D materials

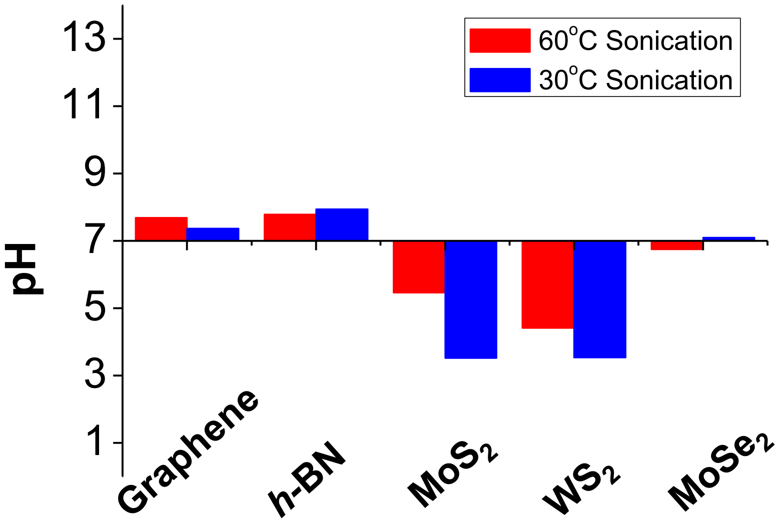

Supplementary Figure 10 pH measurement for five 2D materials at two different sonication temperatures.

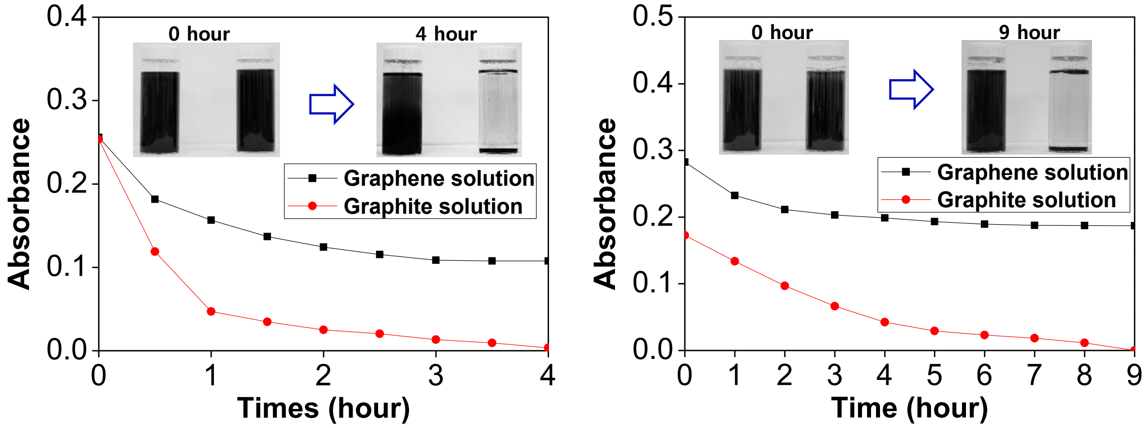

Supplementary Figure 11 Stability comparison of stirred graphite (Black square) and sonicated graphene (red circle). (**a**) low temperature storage and (**b**) high temperature storage. Left bottles are graphene, and right ones graphite.

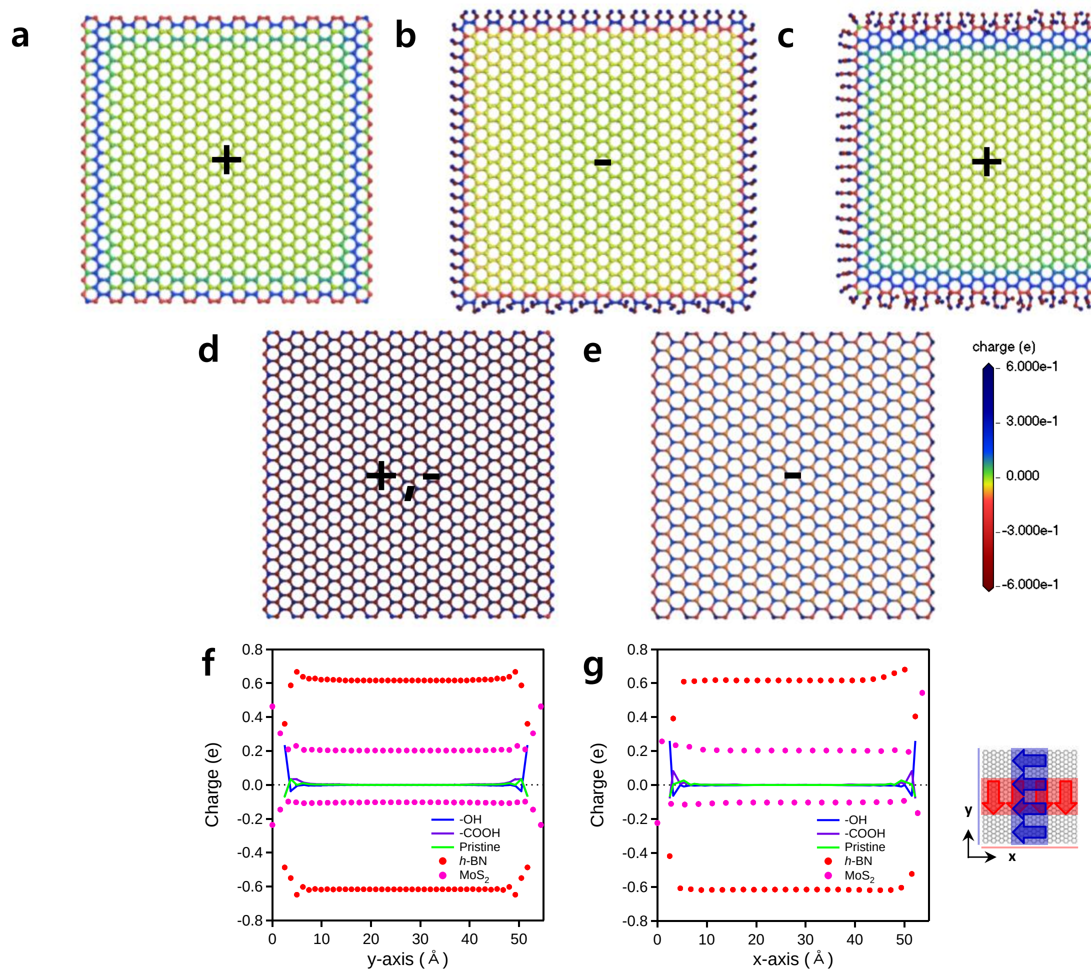

Supplementary Figure 12 Charge distribution information of model nanoparticles. (**a**) pristine graphene, (**b**) 100% -OH functionalized graphene, (**c**) 100% -COOH functionalized graphene, (**d**) *h*-BN, (**e**) MoS_2, (**f**) charge distribution of atoms projected in the armchair direction shown by blue arrows in the right inset, and (**g**) charge distribution of atoms projected in the zigzag direction shown by red arrows in the left inset. The sign symbols '+' or '-' represent surface charges of nanoparticles. For MoS_2, the positive charges are from Mo atoms and the negative ones are from S atoms, and for *h*-BN, boron and nitrogen have alternating charges.

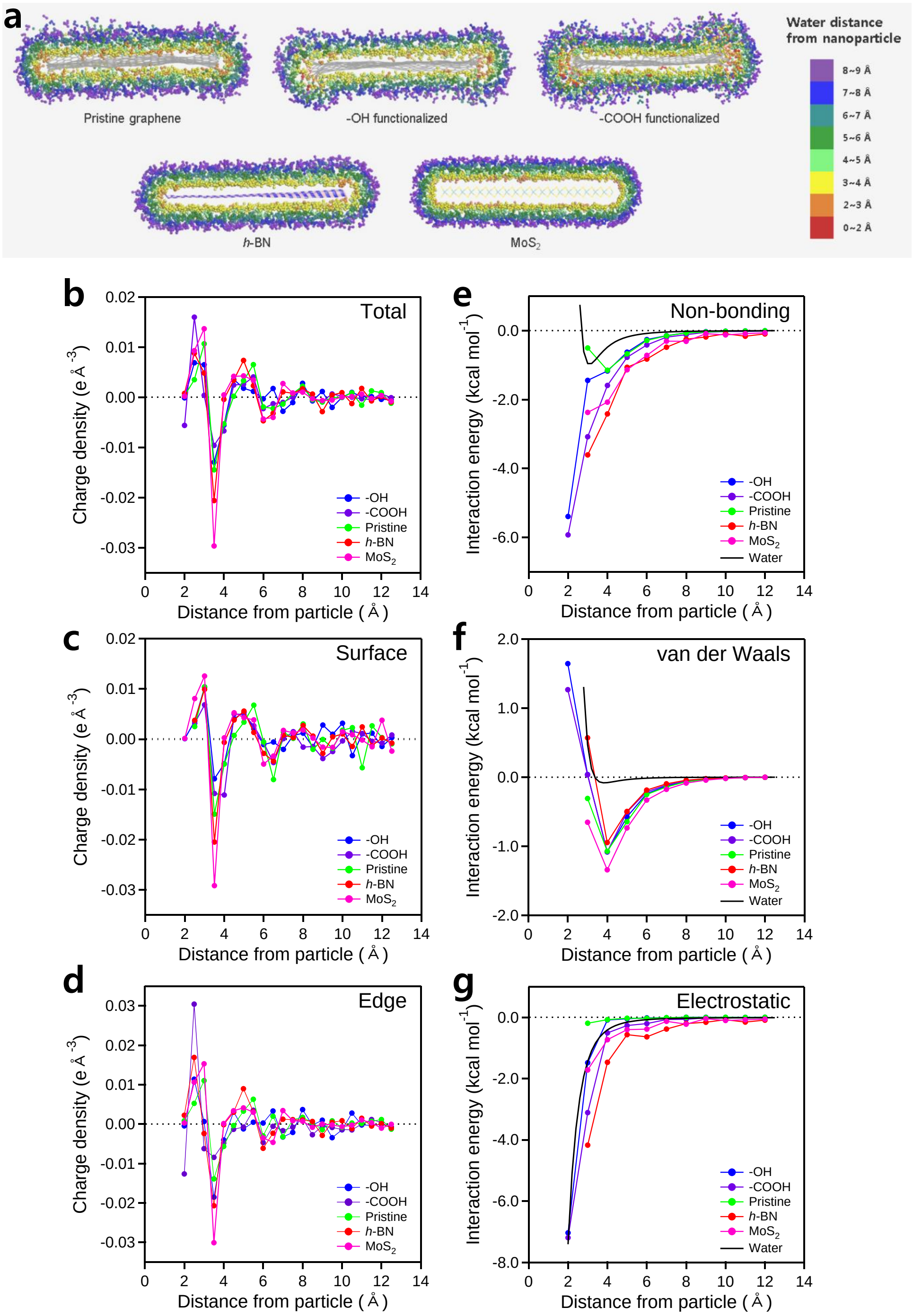

Supplementary Figure 13 Charge density profiles of oxygen and hydrogen atoms near nanoparticles, Water molecule distributions in **a**, are shown in **(b)** to **(d)**. Regions of nanoparticles are separated by 'surface (basal plane)' and 'edge (including functional groups)'. The term 'total' indicates a whole region of nanoparticle. The interaction energies of nanoparticles with a water molecule and a water molecule by a water molecule are shown in **(e)** to **(g)**. The term 'non-bonding' includes van der Waals and electrostatic potential energies. The inset shows the color map of water distribution near nanoparticles in the range of 9Å.

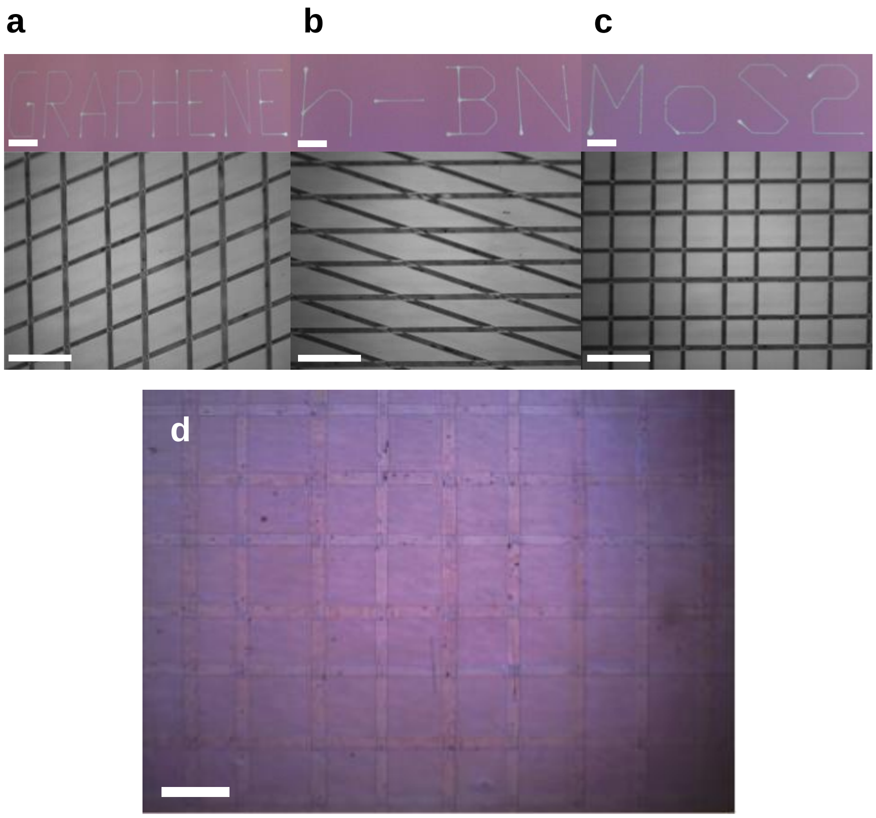

Supplementary Figure 14 Various patterns printed with 2D material inks on hard and soft substrates. Letter and mesh patterns printed on hard substrates with mixed PEO-water inks of (**a**) graphene, (**b**) *h*-BN, and (**c**) MoS_2, (SiO2/Si substrate for letter patterns and Si substrate for mesh patterns) and (**d**) a mesh pattern printed on polyethylene terephthalate (PET) substrate with a mixed PEO-water graphene ink. The scale bars in upper and lower panels of (**a-c**) are 1mm and 200 µm, and the scale bar in (**d**) is 100 µm.

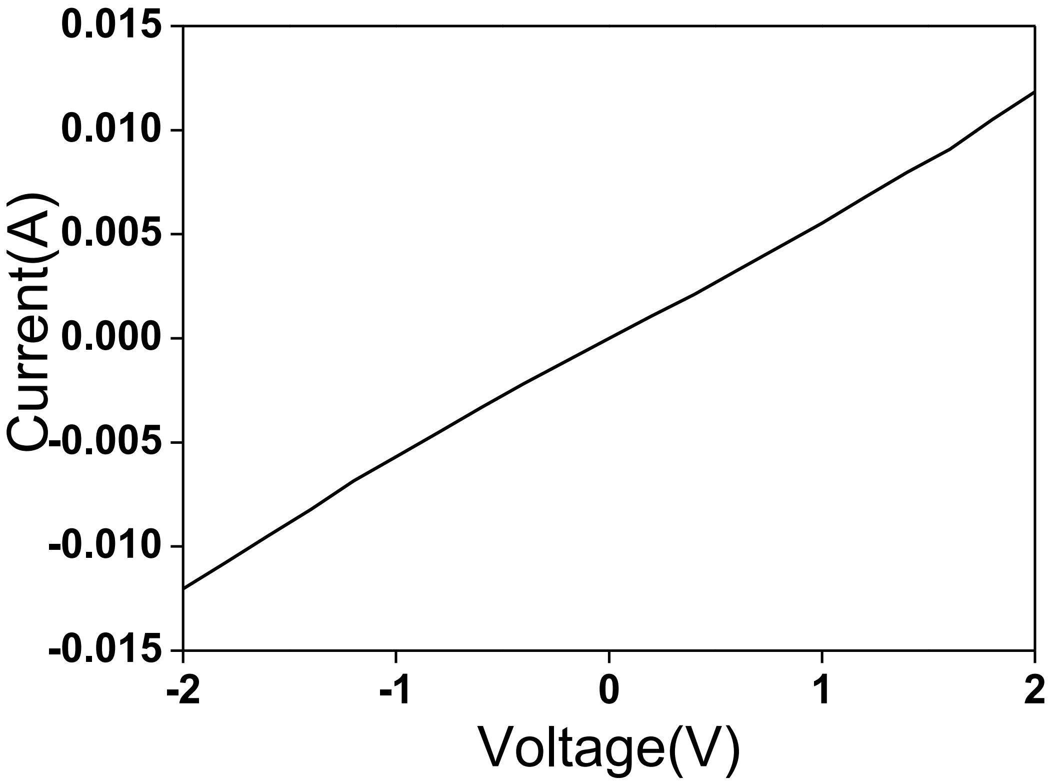

Supplementary Figure 15 Electrical measurement result for graphene film fabricated by filtering graphene-water solution. Thickness of the film is 2.7 μm.

Supplementary Table 1 Comparison of concentration of 2D materials in various solvents.

Solvents	Graphene (mg/ml)	h-BN (mg/ml)	MoS2 (mg/ml)
**Water (this work)**	**0.0065**	**0.018**	**0.13**
**Water/Surfactant**	0.21-0.3[3,4]	N/A	0.25[5]
**NMP**	0.05~1[6]	0.031[7]	0.3[7]
**IPA**	0.5[8]	0.06[7]	0.12[7]
**DMF**	0.0041~0.0045[6,9]	0.024[7]	0.2[7]
**DMSO**	0.0037~0.0041[6,9]	0.36[7]	0.22[7]

Supplementary Table 2 Comparison of flake size of 2D materials in various solvents (lateral size).

Solvents	Graphene (nm)	h-BN (nm)	MoS2 (nm)
**Water (this work)**	200	300	100
**Water/Surfactant**	500[3]	N/A	430[5]
**NMP**	500~3000[6,9]	N/A	N/A
**IPA**	1100[8]	960[7]	170[7]
**DMF**	N/A	N/A	N/A
**DMSO**	N/A	N/A	N/A

Supplementary Table 3 Non-bond potential parameter used for each nanoparticle. Regions of nanoparticles are separated by 'surface (basal plane)' and 'edge (excluding functional groups)'. Energy(ε) and distance(σ) values are in eV, Å, respectively. Partial charges are estimated by Mulliken population analysis.

	Lennard-Jones potential parameters[a]		Partial charges (*e*)	
	ε, σ		surface	edge
Pristine graphene	COMPASS	C	0.000~0.016	-0.089~0.098
-OH functionalized graphene	COMPASS	C	-0.006~0.000	-0.091~0.274
-COOH functionalized graphene	COMPASS	C	0.000~0.016	-0.09~0.115
h-BN	Dreiding	B	0.612~0.681	0.212~0.750
		N	-0.623~-0.599	-0.671~-0.272
MoS_2	Morita *et al.*			
Mo-Mo	0.8382, 2.5510	Mo	0.200~0.236	0.195~0.614
S-S (intra-plane)	0.0019, 3.3695			
S-S (inter-plane)	0.0019, 3.3695	S	-0.119~-0.100	-0.289~-0.093
Mo-S	0.0399, 2.9318			

- [a] LJ potential : $4\varepsilon\left[\left(\frac{\sigma}{r}\right)^{12}-\left(\frac{\sigma}{r}\right)^{6}\right]$

Supplementary References

1 Kumar, P. V. *et al.* Scalable enhancement of graphene oxide properties by thermally driven phase transformation. *Nature chemistry* **6**, 151-158 (2014).

2 Silverstein, R. M. *Spectrometric identification of organic compounds*. (Wiley Online Library).

3 Lotya, M., King, P. J., Khan, U., De, S. & Coleman, J. N. High-Concentration, Surfactant-Stabilized Graphene Dispersions. *ACS Nano* **4**, 3155-3162, doi:10.1021/nn1005304 (2010).

4 Yi, M., Shen, Z., Zhang, X. & Ma, S. Achieving concentrated graphene dispersions in water/acetone mixtures by the strategy of tailoring Hansen solubility parameters. *Journal of Physics D: Applied Physics* **46**, 025301 (2013).

5 Smith, R. J. *et al.* Large-Scale Exfoliation of Inorganic Layered Compounds in Aqueous Surfactant Solutions. *Advanced materials* **23**, 3944-3948 (2011).

6 Coleman, J. N. Liquid Exfoliation of Defect-Free Graphene. *Accounts of Chemical Research* **46**, 14-22, doi:10.1021/ar300009f (2013).

7 Coleman, J. N. *et al.* Two-dimensional nanosheets produced by liquid exfoliation of layered materials. *Science* **331**, 568-571, doi:10.1126/science.1194975 (2011).

8 O'Neill, A., Khan, U., Nirmalraj, P. N., Boland, J. & Coleman, J. N. Graphene dispersion and exfoliation in low boiling point solvents. *The Journal of Physical Chemistry C* **115**, 5422-5428 (2011).

9 Hernandez, Y. *et al.* High-yield production of graphene by liquid-phase exfoliation of graphite. *Nat Nano* **3**, 563-568, doi:http://www.nature.com/nnano/journal/v3/n9/suppinfo/nnano.2008.215_S1.html (2008).
